# Evaluation of a nanophosphor lateral-flow assay for self-testing for herpes simplex virus type 2 seropositivity

**DOI:** 10.1371/journal.pone.0225365

**Published:** 2019-12-10

**Authors:** Heather J. Goux, Balakrishnan Raja, Katerina Kourentzi, João R. C. Trabuco, Binh V. Vu, Andrew S. Paterson, Alexander Kirkpatrick, Blane Townsend, Miles Lee, Van Thi Thanh Truong, Claudia Pedroza, Richard C. Willson

**Affiliations:** 1 Department of Biology and Biochemistry, University of Houston, Houston, Texas, United States of America; 2 Luminostics, Inc., San Jose, California, United States of America; 3 Department of Chemical and Biomolecular Engineering, University of Houston, Houston, Texas, United States of America; 4 Medical School Center for Clinical Research and Evidence-Based Medicine, University of Texas Health Science Center at Houston, Houston, Texas, United States of America; Albert Einstein College of Medicine, UNITED STATES

## Abstract

Herpes Simplex Virus Type 2 (HSV-2) is a common human pathogen that causes life-long illness. The US prevalence of HSV-2 infection is 11.9% for individuals between 15 and 49 years of age. Individuals with HSV-2 infection are more likely to contract and spread other sexually-transmitted infections. Eighty percent of individuals with HSV-2 are unaware of their infection, in part because of the social stigma associated with in-clinic testing for sexually-transmitted infections. We conducted an initial evaluation of a prototype smartphone-based serological lateral-flow immunoassay (LFA) for HSV-2 infection that uses strontium aluminate persistent luminescent nanoparticles (nanophosphors) as reporters. When applied to a test panel of 21 human plasma/serum samples varying in anti-HSV titer, the nanophosphor HSV-2 LFA had 96.7% sensitivity and 100% specificity for detection of HSV-2 infection. The sensitivity of the nanophosphor HSV-2 LFA was higher than that of commercially-available rapid HSV-2 assays tested with the same panel. Analysis of the iPhone nanophosphor HSV-2 LFA strip images with our custom smartphone app gave greater reproducibility compared to ImageJ analysis of strip images. The smartphone-based nanophosphor LFA technology shows promise for private self-testing for sexually-transmitted infections (STI).

## Introduction

The National Health and Nutrition Examination Survey estimates an 11.9% prevalence of HSV-2 in the US population aged 14 to 49 [1]. HSV-2 is the primary cause of genital ulcer disease [2], which causes inflammation and lesions of the genitalia, hence facilitating the entry of other potentially life-threatening sexually-transmitted pathogens [3–7].

The transmission of HSV-2 occurs during viral shedding [8,9], which can precede the appearance of genital lesions [10]. Episodes of shedding continue throughout the lives of infected individuals alongside symptomatic and asymptomatic outbreaks [11]. Preventative measures such as a regimen of the antiviral drug valacyclovir [12,13] and the use of condoms, can drastically reduce the transmission of HSV-2 [14,15]. Despite the high prevalence of HSV-2 most infected individuals are asymptomatic, and only 10–25% of HSV-2 seropositive individuals report the development of genital lesions [15, 16]. Furthermore, high-risk asymptomatic individuals often refrain from in-clinic testing due to the high costs and social stigma associated with being tested. As a result, only 10% of HSV-2 cases are diagnosed clinically [16]. Thus, there is a need for a discreet method of routine self-testing for HSV-2, especially for high-risk populations.

Serology is the preferred method of HSV-2 testing because it enables diagnosis of asymptomatic or latent infections [17–20]. The HerpeSelect 2 assay (Focus Diagnostics, Cypress, CA) is a commonly-used commercial test performed in a traditional 96-well enzyme-linked immunosorbent assay (ELISA) format, which requires both technical expertise and a laboratory setting. Lateral-flow immunoassays (LFAs; the home pregnancy test format) are faster, simpler, portable, and more accessible than ELISA-based assays; and therefore are better suited for home-based self-testing [21]. LFAs are conventionally run with colloidal gold or latex microsphere reporters that produce signals which can be observed by the naked eye, eliminating the need for equipment and making self-testing feasible [22,23].

Currently, there are no commercially available gold nanoparticle LFAs for HSV-2. However, Laderman et al. described the development of a gold nanoparticle-based LFIA serological HSV-2 test and showed that the sensitivity and specificity of the gold LFIA, in reference to HerpeSelect ELISA HSV-2, performed equivalently to the FDA cleared POCkit Test (now referred to as the Sure-Vue HSV-2 Rapid Test)[24]. The Sure-Vue HSV-2 Rapid test, an immunoblot, has been extensively tested in the field to show a sensitivity of 94% and specificity of 98%. If there was a rapid HSV-2 assay available with a higher clinical sensitivity and equivalent or higher specificity, it could potentially be used to screen high-risk populations for HSV-2 seropositivity, reducing the number of undiagnosed (asymptomatic) patients and the transmission of HSV-2. Instrument-based reporters such as up-converting phosphors, magnetic microparticles, and fluorescent labels support more analytically-sensitive LFAs but require expensive components such as intense light sources, magnetic readers, or precision optical filters [25]. We have developed strontium aluminate persistent luminescent nanoparticles (PLNPs, nanophosphors) as LFA reporters [26] whose long-lasting, bright glow after excitation allows for a delay of emission measurement, reducing background autofluorescence and eliminating the need for precision optical filters. Strontium aluminate doped with europium and dysprosium (SrAl_2_O_4_: Eu^2+^, Dy^3+^) has a bright and long-lasting light emission (10X brighter than common ZnS phosphors) and is inexpensive; it is widely used in “glow-in-the-dark” signs and toys. Strontium aluminate PLNP LFAs have been shown to have higher analytical sensitivity than traditional LFAs [27,28], and so may have higher clinical sensitivity. A smartphone app, originally developed at University of Houston [28] and since refined by Luminostics, improves test accuracy by automating LFA signal readout and image analysis, thus eliminating human error and bias. However, previous studies with PLNPs have not demonstrated their use in real human samples for evaluation of clinical sensitivity and specificity.

The purpose of this study is to demonstrate how the high analytical sensitivity inherent to PLNPs and the smartphone platform translates to high clinical sensitivity and specificity in real samples. To do so, we first developed a smartphone-based PLNP HSV-2 LFA and then using a commercial plasma/serum HSV-1/2 panel as a benchmark we characterized and evaluated the performance of the model smartphone-based PLNP LFA and compared it to two commercially available HSV-2 rapid assays.

## Materials and methods

### Clinical samples

A panel of 21 human plasma and serum samples ranging from negative to strongly positive for HSV-1 and HSV-2 (PTH202; SeraCare Life Science; Gaithersburg, MD) was used to characterize the HSV-2 PLNP LFA. Samples were stored -20 °C according to the manufacturer's recommendations.

### Preparation of PLNP

Procedures for grinding, sizing, silica-coating, and antibody-conjugation of strontium aluminate PLNPs were as described previously [26, 28], with minor modifications as noted below. 150 g of strontium aluminate powder (Ultra Green V10 Glow in the Dark Powder, Glow, Inc.) was suspended in 0.5 L ethyl acetate and wet-milled for 24 h in a SWECO Vibro-Energy Grinding Mill (model M18-5; Florence, KY) using 0.5 kg of ¼ inch (6.35 mm) magnesia-stabilized zirconia grinding cylinders. Ethyl acetate was evaporated from the particle suspension while avoiding accumulation of flammable vapors, and differential centrifugal sedimentation in ethanol was used to selectively isolate 200–300 nm nanophosphors [26]. The final concentration of particles was calculated from dried mass measured on an analytical balance (model no. XS64, Mettler Toledo). The fractionated PLNPs were silica-encapsulated with tetraethyl orthosilicate (TEOS) (Sigma Aldrich, cat no. 131903; St. Louis, MO) to increase their water stability using a modified Stöber process [26]. Aldehydes were introduced onto the silica-encapsulated PLNPs using triethoxysilylbutyraldehyde (TESBA) (Gelest, Inc., cat no. SIT8185.3; Morrisville, PA). A 10 μl of a solution containing 10% (v/v) TEOS, 0.3% TESBA, and 89.7% absolute ethanol was added to 784 μl of 1.28 mg/ml silica encapsulated PLNPs suspended in absolute ethanol. The mixture was vortexed and added to 206 μl of a solution of 2.4% NH_4_OH and 97.6% deionized water. After 10 min of sonication on a Fisher Scientific FS30 Ultrasonic Cleaner, the mixture was placed on a rotator at room temperature (RT). After a 12-h silanization period the particles were washed thrice in 1 ml of absolute ethanol, once in 1 ml deionized water, and once in 1 ml PBS (pH 7.4; Takara Bio USA Inc., cat no. T9181; 110 Mountain View, CA). Each wash included collecting the settled particles by centrifugation (3,000 x g for 2.5 min) and resuspending the particles in 1 ml by briefly vortexing and then bath sonicating the PLNP suspension for 3 min. Surface aldehydes on the PLNPs were coupled to primary amine groups on goat anti-human polyclonal antibodies using reductive amination as follows. The particles were centrifuged at 3,000 x g for 2.5 min, resuspended in 1 ml PBS, and incubated with 50 μg goat anti-human IgG polyclonal antibody (Arista Biologicals, Inc., cat no. ABIGG-0500; Allentown, PA) and 250 mM NaBH_3_CN (Chem-Impex International, Inc., cat no. 04836; Wood Dale, IL) at room temperature (RT) on a rotator for 2 h. The particles were washed in 1 ml PBS and resuspended in 200 μl PBS to remove unbound antibodies. To passivate the particles, 800 μl passivation buffer (0.6 mM BSA and 50 μM NaBH₃CN in PBS) was added to the particle suspension and incubated for 3 h on a rotator at RT. The functionalized particles were washed three times in PBS and resuspended at a concentration of 5 mg/ml in 200 μl storage buffer (10 mM BNa_3_O_3_, 150 mM NaCl, 0.1% BSA, 0.4% PVP-40, 0.025% Tween 20, pH 8.5). All chemicals and detergents in the storage buffer were purchased from Sigma and were of analytical grade.

### LFA strip preparation

**Fig 1:**
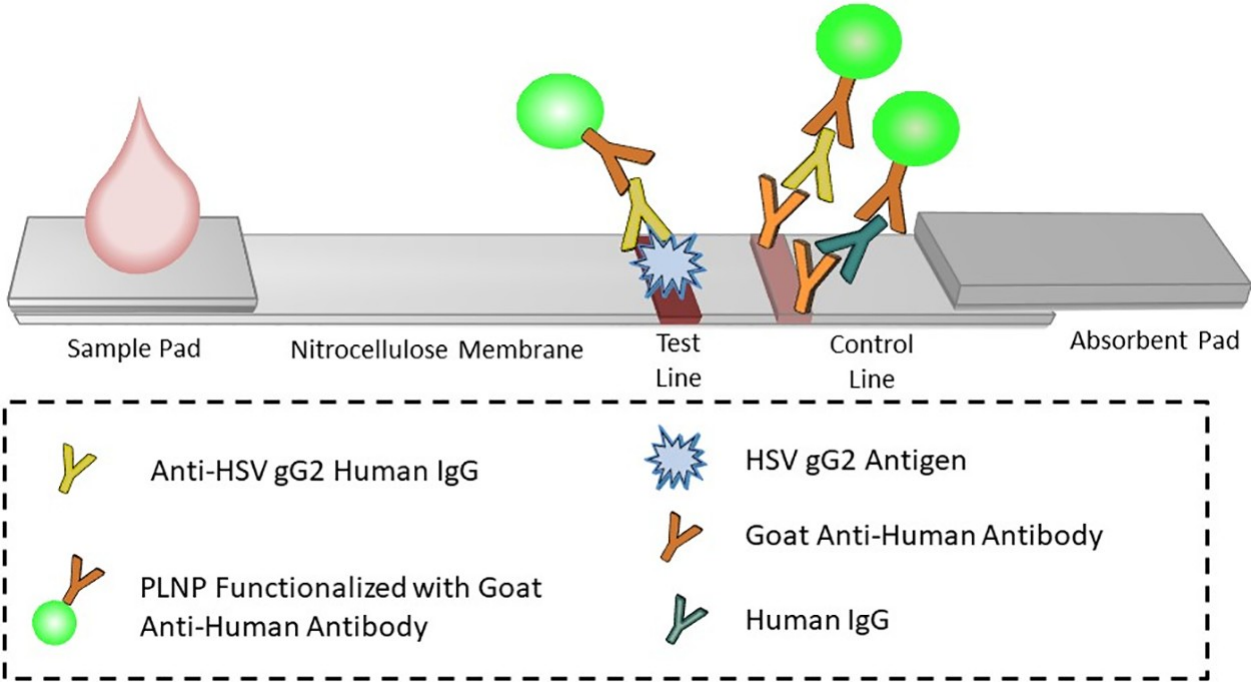
HSV-2 PLNP LFA schematic. A sample is diluted and then mixed with PLNPs functionalized with goat anti-human IgGs to form human IgG-PLNP complexes that were dispensed onto the sample pad of the LFA strip. The anti-HSV human IgG PLNP complexes migrated up the membrane and were captured by recombinant HSV gG2 immobilized at the test line. The remaining uncaptured human IgG-PLNP complexes, whether specific to HSV2 gG2 or not, continued further up the strip until they were captured by goat anti-human IgGs immobilized at the control line.

LFA strips were composed of a Sartorius Stedim Biotech UniSart CN95 backed nitrocellulose membrane (30 mm × 3 mm; Bohemia, NY), a Whatman GE Healthcare Standard 14 sample pad (11 mm × 3 mm; Pittsburgh, PA), a Whatman GE Healthcare Standard CF5 absorbent pad (22 mm × 3 mm), and an adhesive backing card (DCN Diagnostics, cat no. MIBA-020; Carlsbad, CA), as shown in Fig 1. Test and control lines were generated using a Lateral Flow Reagent Dispenser (Claremont BioSolutions; Upland, CA) and a Fusion 200 syringe pump (Chemyx, Inc.; Stafford, Tx) at a dispense rate of 0.22 ml/min and a head speed of 4 cm/s. To make the control line, 1 mg/ml goat anti-human IgG capture antibody (Arista Biologicals, Inc., cat no. ABIGG-0500; Allentown, PA) in 100 mM ammonium acetate, pH 5.4 was dispensed onto the membrane 2 mm from the absorbent pad. To make the test line, 425 μg/ml recombinant HSV gG2 antigen (BioSpacific, Inc., cat no. RAG0087; Emeryville, CA) in 100 mM ammonium acetate at pH 5.4 was dispensed onto the membrane 7 mm from the absorbent pad. The sample pad was soaked in blocking buffer (10 mM Tris-HCl, 0.5% PVP-40, 0.05% Tween 20, 1% BSA, pH 8) for 1 h and then blotted and dried at RT overnight. The assembled LFA materials were cut using a ZQ2000 Guillotine Cutter (Diagnostic Tech Co., Shanghai, China) into 3 mm wide LFA strips.

### HSV-2 PLNP LFA

10 μl human plasma or serum samples were diluted in 25 μl of buffer (10 mM Tris-HCl, 5% Tween 20, pH 8.0). 20 μl of the diluted sample was mixed with 15 μl anti-human IgG-PLNP conjugate [1.5 μg PLNP functionalized with goat anti-human IgG suspended in running buffer (10 mM Tris-HCl, 50 mM NaCl, 0.1% Tween 20, 0.25% PVP-40, pH 8)]. 35 μl of the sample/nanophosphor mixture was dispensed onto the LFA sample pad (Fig 1). Goat anti-human IgG nanophosphors bearing anti-HSV human IgG, if present in the sample, migrated up the membrane and were captured by the recombinant HSV gG2 antigen immobilized at the test line. Unbound anti-human nanophosphors bearing human IgG migrated further up the strip until they were captured by the goat anti-human IgGs immobilized at the control line.

### Imaging of the LFA strips

**Fig 2:**
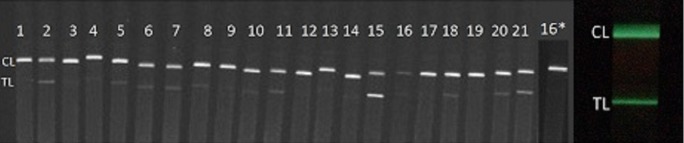
Representative images of the HSV PTH202 serum/plasma panel tested with the HSV-2 PLNP LFA. The strips were imaged as a group on the FluorChem Platform (left side, panel members 1 to 21) or individually on the iPhone platform (right, panel member 2). The images capture a region that encompasses both the test line (TL) and control line (CL) on the test strip. Panel member 16 consistently showed a significantly weaker control line which was undetectable by the smartphone. For further investigation, Panel member 16 was re-tested at a higher dilution (20-fold instead of 3.5-fold), and the picture was added to the panel image (denoted by the asterisk).

LFA test strips were imaged on a FluorChem-based imaging platform composed of a FluorChem SP gel cabinet (Alpha Innotech Corp., San Leandro CA), two 10 W ultraviolet LED lights (wavelength: 395–400 nm, forward voltage: 9V–11V, forward current: 900 mA; LED World), and a CoolSNAP K4 CCD 2,048 × 2,048-pixel camera (Photometrics, Blaine WA) controlled by Micro-Manager 1.4.22 software (Vale Lab, University of California, San Francisco). PLNP were excited with the LEDs for 1 min and imaged with an exposure time of 1 s and pixel binning of 4 (Fig 2, left).

LFA test strips also were imaged on an iPhone 7 Plus Smartphone (Apple Inc., Cupertino, CA) using a custom LFA iPhone app in combination with a 3D-printed attachment, as described previously [28]. PLNPs were excited by turning on the iPhone torch (4 s, maximum intensity) and then cycling the camera’s flash. After a delay *ca*. 100 ms after excitation, an image of phosphor emission was acquired with the phone camera. The excitation/imaging cycle was repeated four times, and the four images were stacked together to reduce background noise and increase reproducibility (Fig 2, right).

### Image processing

ImageJ version 1.8.0_112 (U. S. National Institutes of Health; Bethesda, MD) [29] was used to analyze both Fluorchem Platform and iPhone strip images. Before measurement, a spatial calibration was performed to set the number of pixels across the width of the LFA strip (3 mm), and a plot profile of pixel intensities as a function of position along the membrane was generated. A line was drawn across the base of each peak on the graph to allow its area to be integrated. The areas of the peaks located at the test line and the control line were defined as the test line signal (TL) and the control line signal (CL), respectively. We calculated the relative signal of the test line (T/C) as the ratio of TL to CL (S5 Fig).

The iPhone strip images were also automatically analyzed using the LFA app (video at https://drive.google.com/file/d/18cdR—1s9s-LcqjvvXjCBtQ_DRmv76Wu/view?ts=5cc9ebce). The app locates the inflection points on the peaks in the pixel intensity profiles of the captured bright-field RGB image that correspond to the edges of the test and control lines; the pixel intensities within the enclosed regions are averaged to give the effective intensities of the test line and control line. Only green pixels on the RGB image are used in the calculations because SrAl_2_O_4_: Eu^2+^, Dy^3+^ has a green emission peak at 520 nm that most strongly overlaps with the green channel. The baseline intensity (average pixel intensity of the region between the test and control peaks) is then subtracted from the average pixel intensity at the test and control lines to calculate the TL and CL intensities (S5 Fig). 4 of 63 tests produced strip images with a TL intensity either below or above the quantifiable range of the LFA app. These images were inspected visually and classified as a strong negative (1 test) or strong positives (3 tests); this function will be automated in future versions of the software.

### Total IgG ELISA

A Maxisorp 96-well ELISA plate (Greiner Bio-One) was incubated overnight at 4°C with 5 μg/ml polyclonal goat anti-human IgG in PBS (100 μl per well). The liquid was removed and wells blocked with 3% BSA, 0.1% fish gelatin, and 3 mM EDTA in PBS for 2 h at RT (300 μl per well). The plate was then washed three times with PBS containing 0.1% Tween 20 using a Tecan Hydroflex plate washer. A 12 mg/ml stock solution of human IgG standard (Arista Biologicals, Inc., cat no. AGHIG-0100; Allentown, PA) was diluted in PBS containing 3 mM EDTA, 2% BSA, and 0.05% Tween 20 to prepare standards ranging from 30 to 0.001 μg/ml IgG. PTH202 panel samples were diluted 10,000-fold and 100,000-fold in dilution buffer. 100 μl of standards (n = 3) and sample dilutions (n = 1) were added to the plate and incubated at RT for 2 h, and the wells were emptied and washed five times with PBS with 0.1% Tween 20. 100 μl of 100 ng/ml anti-human IgG (γ-chain)-HRP conjugate (Sigma-Aldrich, cat no. A6029; St. Louis, MO) in PBS containing 1% BSA, 0.1% gelatin, and 0.05% Tween 20 was added to each well and incubated for 1 h at RT. The plate was washed three times with PBS with 0.1% Tween 20. 100 μL 1-Step Ultra TMB ELISA Substrate Solution (Thermo Fisher Scientific, Inc., cat. no. 34028; Rockford, IL) was added to each well. Following incubation for 10 min at RT, the reaction was stopped with 2M H_2_SO_4_ (50 μL), and the absorbance was measured at 450 nm using a Tecan Infinite M200 Pro plate reader. The absorbance of each standard was plotted against IgG concentration (n = 3), and the data were fit to a four-parameter logistic curve using the MyAssays ELISA analysis online tool (MyAssays Ltd., www.myassays.com/four-parameter-logistic-curve.assay; S3A Fig). The IgG concentration of panel member 17 had a mean absorbance that could not be accurately measured in ELISA, as it fell above the quantifiable range of standard curve. Thus, it is to be expected that member 17 has a total IgG concentration greater than 23.3 mg/ml (the highest quantifiable concentration).

### PTH202 Mixed Titer Performance Panel testing on alternative immunoassay platforms

SeraCare tested their PTH202 Mixed Titer Performance Panel with 12 different commercial HSV-1 or HSV-2 immunoassays; all raw data and test results from these experiments can be accessed online (www.seracare.com/globalassets/seracare-resources/ds-0815-0003-anti-herpes-mixed-titer-performance-panel-124412.PDF). We used this data to evaluate and compare the performance of the PLNP HSV-2 LFA to other rapid assays.

Data from panel testing on HerpeSelect 2 ELISA (the reference method) was used to determine the sensitivity and specificity of the PLNP HSV-2 LFA. SeraCare calculated PTH202 panel HerpeSelect 2 immunoassay results as the mean (n = 2) of signal-to-cutoff ratios (reactivity ratios) for each panel member. SeraCare’s reported specifications considered panel members with average reactivity ratios < 1.0 and ≥1.0 as negative and positive, respectively, for human IgG antibodies specific to HSV-2.

The HerpeSelect 2 ELISA (Focus Diagnostics, Cypress, CA) is an FDA-cleared test that determines the presence of HSV gG-2-specific IgGs in human serum/plasma. The HerpeSelect 2 ELISA demonstrated the highest sensitivity in a comparison among five of the leading commercial HSV-2 assays [17]. Using the CDC’s gold standard method (HSV western blot from the University of Washington [30,31]) as a reference, the HerpeSelect 2 ELISA had 96.1% sensitivity and 97.0% specificity in the testing of sexually active adults [32,33].

### Statistical analysis

Receiver operating characteristic (ROC) curves, which depict the relationship between the fraction of positive samples correctly identified as positive (sensitivity) and false-positive results (1- specificity) for various cutoff values, were constructed using MedCalc software version 18.2.1 (MedCalc Software; www.medcalc.org; Ostend, Belgium). Each sample was tested three times using the HSV-2 PLNP LFA (n = 63). A TL above or below the range of quantitation of the iPhone app was given a value of 71 (the upper limit of quantitation) or 0, respectively. One sample, number 16, showed no control line on the iPhone (though the CL was visible on the laboratory FluorChem Platform). After successfully re-testing sample 16 when diluted 20-fold (and observed the control line; Fig 2, strip 16*), we speculated that the earlier result was due to the complex sample matrix, along with its high IgG concentration. Each panel member was defined as positive or negative in reference to the HerpeSelect 2 ELISA, the Reference Method.

## Results and discussion

We developed an LFA to detect the presence of human IgG antibodies specific to HSV-2 in serum or plasma. The assay uses a test line of recombinant glycoprotein G from HSV-2, a control line of goat anti-human antibodies, and strontium aluminate PLNPs modified with goat anti-human antibodies as reporters. We evaluated the HSV-2 PLNP LFA using a 21-member panel with a wide range of mixed HSV-1 and HSV-2 antibody titers. We captured images of the test strips on both the FluorChem Platform (S1 Fig) and an iPhone 7 Plus-based platform. We then analyzed both FluorChem Platform and iPhone images with ImageJ, and also analyzed the iPhone images with a PLNP LFA iPhone app. We used the resulting measurements to evaluate the performance of the HSV-2 PLNP LFA.

### Defining and evaluating the function of the HSV-2 PLNP LFA control line

As noted above, the HSV-2 PLNP LFA was developed with the eventual goal of use in a home setting by an inexperienced user. Traditional LFA control lines function by capturing reporter particles directly to confirm that the liquid sample has wicked along the strip (e.g., the absence of a control line indicates an invalid test in home pregnancy LFAs). We designed the control line in the HSV-2 PLNP LFA to function differently. Here, the control line (consisting of immobilized anti-human IgG antibodies) captures anti-human reporter particles bearing human antibodies from the sample, instead of directly capturing the reporter particles in the absence of target. This design not only confirms that the liquid sample has wicked along the strip but also that the correct sample type has been applied onto the strip; only human blood containing human antibodies will give a positive CL. As expected, no control line was detected using with either deionized water or PBS (S4 Fig), but detectable control lines were obtained with typical adult human serum/plasma samples.

IgG concentrations in human blood can vary dramatically (0.4–22.0 mg/ml) depending on age, sex, illness, immunogen exposure, and other factors [33]. The total IgG ELISA results (S3A Fig) showed the PTH202 panel members to range from 5.7 (No. 8) to 23.3 (No. 13) mg/ml of human IgG (S3B Fig). When we tested the PTH202 panel in the HSV-2 PLNP LFA, all but one member (No. 16) produced a CL that was above the detectable analytical limit of the smartphone app. Therefore, we can conclude that the PLNP LFA control line was able to detect samples that range from 5.7 to 23.3 mg/ml in human IgG concentration. We also can conclude that we can detect beyond the normal upper IgG concentration in human serum or plasma (22.0 mg/ml).

The total IgG ELISA showed that while No. 16 has a high IgG concentration (23.1 mg/ml; S3B Fig), No. 13 is even higher at 23.3 mg/ml and still produced a CL detectable by the PLNP LFA iPhone app (both 13 and 16 CLs were detectable by the FluorChem imaging system). We hypothesize, therefore, that sample 16 had a complex sample matrix that interfered with binding at the control line. Running the sample at a higher dilution reduced the sample’s viscosity along with its IgG concentration, increasing reporter mobility and downstream binding at the CL. Sample No. 16 was serially diluted (2- to 20-fold) and was retested with the HSV-2 PLNP LFA. FluorChem images and ImageJ analysis showed that the CL of the 20-fold diluted sample was 5 times greater than that of the undiluted sample (S2 Fig). Therefore, we speculate that the complex sample matrix of panel member 16, along with its high IgG concentration, led to the absence of a CL in the iPhone images. Despite the variation of IgG concentration within the panel (S3B Fig) shown by the total IgG ELISA, LFA tests showed a highly similar CL across the panel (S3C Fig), suggesting that reporter particle and IgG concentrations are more than high enough to produce a detectable CL.

### Definition of the HSV-2 LFA signal readout

Instrument-read LFAs often normalize the test line signal to the control line signal to reduce variability and increase reliability [13, 34–37]. To examine this effect, the reliability and reproducibility of the HSV-2 PLNP LFA were evaluated in terms of the intraclass correlation coefficient (ICC) and the coefficient of variation (CV), respectively, when the assay signal was defined as TL or T/C (TL/CL). HSV-2 PLNP LFA strips were analyzed with different combinations of imaging devices (FluorChem Platform or iPhone camera) and image analysis tools (Image J or app). For Method A (Table 1), the average TL and T/C values were calculated for each PTH202 panel member (n = 3, S1 Table). The same calculations were then repeated for both Methods B and C.

**Table 1:** Comparison of single measure intraclass correlations (ICC) in a two-way mixed-effects model for consistency of agreement using different combinations of imaging devices and image analysis tools (method A, B, C) for the same set of LFA strips. For each method, the ICC is followed by the 95 percent confidence interval enclosed in brackets.

Method	Camera	Image Analysis	Test Line (TL) ICC	Relative Signal (T/C) ICC
A (n = 21)	FluorChem Platform	ImageJ	**0.96** (0.92–0.98)	**0.97** (0.93–0.99)
B (n = 21)	iPhone camera	ImageJ	**0.91** (0.82–0.96)	**0.95** (0.91–0.98)
C (n = 18)	iPhone camera	iPhone app	**0.80** (0.62–0.91)	**0.82** (0.65–0.92)

ICC values were calculated in STATA Statistical software version 14 (StataCorp LPS, 2015; College Station, TX) using a two-way mixed effects model for consistency of agreement. Reporting ICC as single or an average measure depends on whether, in the actual application of the method, the final test result is based on a single measure or an average of multiple measurements. We chose to report ICC values as single measurements because in a home-based setting a single test is more realistic. In this paper, methods that produced ICC values below 0.5 were considered to have poor reliability, values between 0.5 and 0.75 were considered to have moderate reliability, values between 0.75 and 0.9 were considered to have good reliability, and values above 0.9 had excellent reliability [34]. As shown in Table 1, both TL and T/C values showed excellent reliability when measured with strips imaged by FluorChem Platform/images analyzed by ImageJ (Method A). Strips imaged by the iPhone camera/images analyzed by ImageJ (Table 1, Method B) produced good reliability for TL and excellent reliability for T/C. Both TL and T/C values had good to moderate reliability with strips imaged by the iPhone camera/images analyzed by the app (Table 1, Method C). Three panel members produced one or more tests with TL or CL signals above or below the quantifiable range of the iPhone app and thus were omitted when analyzed using Method C (Table 1). Specifically, panel members 21 (one test) and 15 (two tests) had TLs above the quantifiable range. Panel member 16 had TLs (two tests) and CLs (all three tests) below the quantifiable range.

Variability of T or T/C was expressed as CV, the ratio of the standard deviation of the signal to the mean signal. For Methods A and B, the all-sample average CV was 40% and 41% for TL and 45% and 41% for T/C, respectively. Smartphone-based imaging and analysis (Method C) produced the lowest overall CV of the three methods, 15.0% for TL and 14.9% for T/C, (n = 18) indicating a marked increase in reproducibility when strip images were analyzed with the PLNP LFA smartphone app, which is also the method most representative of home self-testing. ImageJ analysis requires visual inspection to determine TL and CL position, while the smartphone app makes this determination automatically. The manual selection of the baseline can be somewhat subjective, leading to the observed reduction in reproducibility. For all methods of analysis, TL and T/C readouts produced a similar degree of variability. We, therefore, chose to define the HSV-2 PLNP LFA readout solely as the TL. The ICC analysis also confirmed that the smartphone app could be used to analyze the LFA strip images with high reliability and we therefore chose the smartphone platform for all subsequent experiments.

### Determination of the positive cutoff value

**Fig 3:**
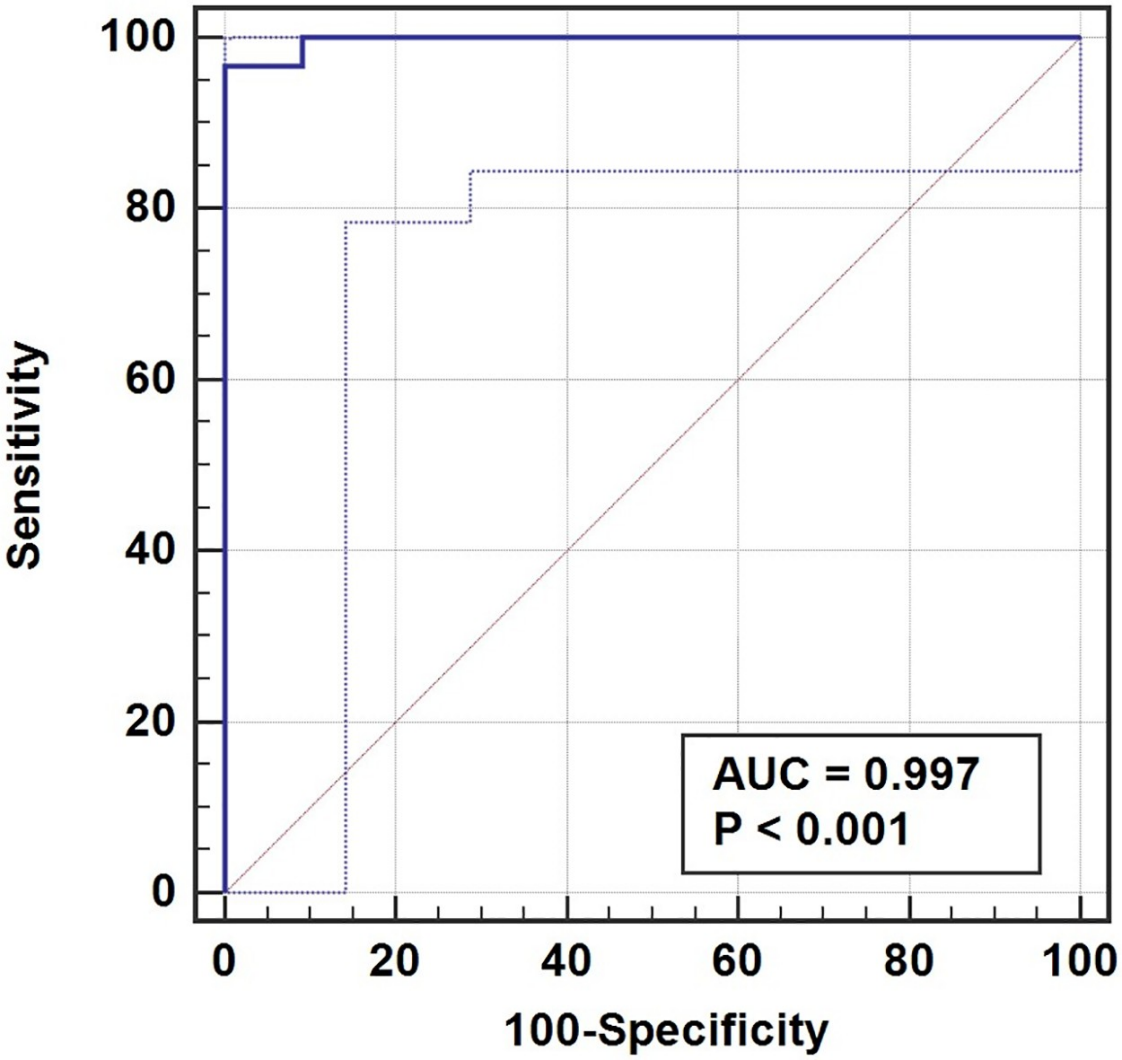
HSV-2 PLNP LFA receiver operating characteristic (ROC) curves. Each panel member was tested and analyzed in replicate (n = 2), by SeraCare, Inc, using HerpeSelect 2 ELISA from Focus Diagnostics, and the resulting absorbance mean was normalized to generate signal-to-cutoff ratios (reactivity ratios). Following SeraCare’s reported specifications, we considered panel members with average reactivity ratios < 1.0 and ≥1.0 as negative and positive, respectively, for human IgG antibodies specific to HSV-2. The panel was tested three times using the HSV-2 PLNP LFA (n = 63) with smartphone-based imaging and app readout. TLs above or below the range of quantitation of the iPhone app were given a value of 71 (the upper limit of quantitation) or 0, respectively. An HSV-2 PLNP LFA receiver operating curve (solid dark blue curve) was generated in MedCalc (version 18.2.1) using test line signal and positive cutoff values, to determine a test positive or negative. Each positive or negative test was then defined as true positive, true negative, false positive, or false negative when referenced to the gold standard method, HerpeSelect 2 ELISA. Based on the ROC analysis, a positive cutoff value of 8 for the TL of HSV-2 PLNP LFA produced the highest sensitivity (96.7%) while maintaining 100% specificity. The ROC curve and analysis gave a value of 0.997 for the area under the curve (AUC) with a 95% confidence interval of 0.937 and 1.00. The confidence region for the ROC curve is represented by the area between the light blue dotted lines.

The effects of varied choices of positive cutoff value for the HSV-2 PLNP LFA were investigated using a receiver operating characteristic (ROC) curve (Fig 3). We tested each panel member (n = 21) three times using the HSV-2 PLNP LFA on the smartphone platform, and used each result as an independent measurement (n = 63) in the ROC analysis. Each positive or negative test was then defined as true positive, true negative, false positive, or false negative when referenced to the gold standard method, HerpeSelect 2 ELISA. We defined the true-positive rate (TPR), or assay sensitivity, as the number of true-positives divided by the sum of true-positives and false-negatives (TPR = TPs/ (TPs+FNs)). The false-positive rate (FPR) was equal to the number of false-positives divided by the sum of the false-positives and true-negatives (FPR = FPs/ (TNs+FPs)). We defined assay specificity as one minus the false-positive rate. Based on the ROC analysis, a positive cutoff value of 8 for the TL of HSV-2 PLNP LFA produced the highest sensitivity (96.7%) while maintaining 100% specificity. However, it must be noted that this positive cutoff value may be adjusted to best fit on the testing population.

### Assay reproducibility

To assess the day-to-day reproducibility of the HSV-2 PLNP LFA; all panel members were tested three times, on different days, using smartphone imagine and the app readout. The resulting TLs were then classified as positive or negative using a cutoff value of 8. Out all 21 panel members, only one subject (panel member 18) had varying positive/negative result classification, indicating a 95% reproducibility (20/21). Panel member 18 had an average TL (8.2 +/- 0.6) that fell closest to the positive cutoff value and gave one negative and two positives. By majority, we deemed panel member 18 as a test positive by the smartphone-based HSV-2 PLNP LFA, a result that was in concordance with the reference assay.

### HSV-1 cross-reactivity

Among potential off-target pathogens, HSV-1 is the most likely candidate for cross-reactivity as it has the closest structural similarity to the HSV-2 virus. Furthermore, a lack of cross-reactivity to HSV-1 is essential for HSV-2 diagnosis due to 47.8% prevalence of HSV-1 in the US population [1,35,36]. Any cross-reactivity to HSV-1 would produce false positives, with potential adverse psychological effects on individuals seropositive for HSV-1 and un-infected with HSV-2 [18,37]. To prevent against HSV-1 cross-reactivity, the Herpes PLNP assay was designed with an HSV-2 Gg-2 antigen test line. Numerous previous results establish that the structure of the HSV-2 Gg-2 antigen is highly serotype-specific. To confirm the lack of cross-reactivity to HSV-1, 10 samples that were HerpeSelect HSV-1 IgG ELISA positive and HerpeSelect HSV-2 IgG ELISA (reference method) negative, consistently tested negative for HSV-2 with the HSV-2 PLNP LFA. Any cross-reactivity of the HSV-2 PLNP LFA to other sexually transmitted infections will need to be evaluated in future studies.

### Comparison of sensitivity and specificity among HSV-2 rapid assays

We used prior results obtained with the PTH202 panel to compare the performance of the LFA to those of two other rapid IgG HSV-2 tests: The Fisher Sure-Vue and the Focus HerpeSelect 1 & 2 Immunoblot. For all rapid assays, a positive or negative result for each panel member was based on the mean signal (n = 2 or n = 3; depending on the rapid test). HerpeSelect 2 ELISA was used as the gold standard, as described above. As shown in Table 2, when analyzed this particular panel all the rapid assays showed 100% specificity; while the HSV-2 PLNP LFA showed the highest sensitivity, followed by the Fisher Sure-Vue assay and the Focus HerpeSelect 1 & 2 Immunoblot.

**Table 2:** Comparison of test metrics for HSV- 2 rapid tests based on the Seracare panel PTH202. The results for each assay are based on the average of 2 or 3 replicates.

Rapid HSV-2 Assay	TP	FP	TN	FN	Sensitivity (%)	Specificity (%)
Fisher Sure-Vue HSV-2 Rapid Test	9	0	11	1	90	100
Focus Diagnostics HerpeSelect 1 & 2 Immunoblot IgG	6	0	11	4	60	100
HSV-2 PLNP LFA	10	0	11	0	100	100

## Conclusions

While this is not the first study to introduce nanophosphor LFAs, this is the first nanophosphor LFA study to use actual human clinical samples, and the first to compare to other LFA platforms. Previous papers describe the development and potential advantages of PLNPs as reporters in LFA and a smartphone-based PLNP LFA platform. These publications focused on the underlying chemistry of the PLNPs, methods for making the nanoparticles, and methods for time-gated imaging and signal quantitation of the luminescence on the smartphone. However, these publications did not fully demonstrate a workable PLNP LFA with smartphone-based analysis in real clinical sample matrices, nor do these publications directly evaluate and compare performance of the technology to other commercially available rapid tests. The present study is a critical step towards validating the smartphone-based PLNP LFA platform as a viable contender for enabling facile, highly sensitive point-of-care testing in the real world. This work is needed to fill the gap between a potential tool for quick and convenient protein-based detection and an at home-based self-testing platform for STI.

The purpose of this manuscript is to demonstrate that the high analytical sensitivity inherent to PLNPs and the smartphone platform translates to high clinical sensitivity and specificity in real samples. Human factors are a significant challenge when developing over-the-counter tests for home diagnostics, as untrained users are prone to erroneously interpreting the results for visual LFAs. This is a major part of the reason why many gold nanoparticle LFAs that are widely used in conventional in-clinic point-of-care testing, are not cleared for over-the-counter home testing by the general public. The smartphone platform eliminates subjective result interpretation and enhances sensitivity by incorporating PLNPs. Furthermore, smartphone-based readout offers the potential for adjusting the discrimination threshold along the ROC curve towards an optimal combination of sensitivity and specificity to achieve a targeted positive predictive value and negative predictive value. This capability is particularly important for screening distinct populations where the prevalence may differ significantly.

## Supporting information

S1 Fig Twenty-one serum and plasma samples in the mixed IgG titer HSV-1/2 panel were tested using the HSV2 nanophosphor LFA. LFA strips were then imaged on the FluorChem Platform and analyzed with ImageJ. (A) The average test line intensity values and (B) the average relative intensity values (T/C; test line intensity/control line intensity) were plotted for each member of the panel; n = 3; average ± 1 standard deviation.
(DOCX)

S2 Fig Sample matrix for panel member 16 had an unusual level of interfering components that adversely affected the assay. FluorChem Platform images of HSV-2 PLNP LFA strips applied to panel member 16 diluted 20-fold (left) or 3.5-fold (right). Running the sample at a higher dilution reduced the sample’s viscosity, increasing reporter mobility and downstream binding at the CL.
(DOCX)

S3 Fig The variation of IgG concentration within the panel and its effect on HSV-2 PLNP LFA control line intensity. (A) Four-parameter logistic calibration curve for the total IgG ELISA created with the online MyAssays ELISA analysis tool. The curve was generated from the raw absorbance at 450 nm of dilutions of human IgG standard (30 to 0.001 ug/mL; Arista Biologicals Inc.) run in the total IgG ELISA. The raw absorbance at 450 nm of 10,000 fold dilutions of each panel samples run in the Total IgG ELISA were plotted against the standard curve (A) to calculate the IgG concentration of sample dilutions which were then used to calculate the IgG concentration of the panel samples in units of mg/mL (B). One sample (panel member 17) had a mean absorbance value that fell above the quantifiable range of standard curve and so its IgG concentration could not be accurately measured. Therefore, we excluded panel member 17 from analyses that compared LFA CL intensity with the IgG concentration. (C) FluorChem Platform control line intensity and IgG concentration of HSV2 panel members. Panel members are specified as negative (red) and positive (green) according to both PLNP HSV2 LFA and HerpeSelect 2 ELISA IgG. The sample number is placed next to each data point. Despite the variation of IgG concentration within the panel, LFA tests showed a highly similar CL across the panel, suggesting that reporter particle and IgG concentrations are more than high enough to produce a detectable CL.
(DOCX)

S4 Fig A luminescence image captured on the FluorChem platform of three HSV-2 PLNP LFA test strips; (left) representative positive (No. 21), (middle) 1 mg/ml human IgG representative of a negative test, (right) representative inconclusive test run with DI water. In the left-hand and middle test strips, the control line (immobilized anti-human IgG antibodies) captures anti-human reporter particles bearing human antibodies from the sample (No. 21 or solution of 1 mg/ml of human IgG) to result in a visible control line. The absence of a control line, as seen in the right-hand test strip, is due to the absence of human IgG antibodies in the sample (DI water); the control line cannot capture anti-human reporter particles not bearing human antibodies. This test indicates that the control line is operating properly—only when the correct sample type has been applied onto the strip; only human serum, plasma, or blood containing human antibodies will give a positive CL.
(DOCX)

S5 Fig Peak area measurements of the test and control lines on LFA strips run with each of the 21 panel members (n = 3). Measurements obtained from iPhone 7 Plus imaging and PLNP-LFA Smartphone App analyses (upper table) and FluorChem imaging and analyses (lower table).
(DOCX)

S1 Table Output from STATA analysis for sensitivity/specificity and positive and negative predictive value of two rapid IgG HSV-2 tests (Fisher Sure-Vue and Focus HerpeSelect 1 & 2 Immunoblot). For each test, a positive or negative result for each PTH202 panel member was based on the mean signal intensity (n = 3). We used the HerpeSelect 2 ELISA to determine sample positives (OD 450 ≥ 1; “an abnormal diagnosis”) and sample negatives (OD 450 < 1) and calculate the number of true-positive, true-negative, false-positive, and false-negative results for each test.
(DOCX)
