# Supplementary material for: Evaluation of a nanophosphor lateral-flow assay for self-testing for herpes simplex virus type 2 seropositivity
Source: PLoS One. 2019 Dec 10;14(12):e0225365. doi: 10.1371/journal.pone.0225365 (PMC6903713; doi:10.1371/journal.pone.0225365)
Supplement: S1 Fig — LFA strips were then imaged on the FluorChem Platform and analyzed with ImageJ. (A) The average test line intensity values and (B) the average relative intensity values (T/C; test line intensity/control line intensity) were plotted for each member of the panel; n = 3; average ± 1 standard deviation. (DOCX) [file pone.0225365.s001.docx]

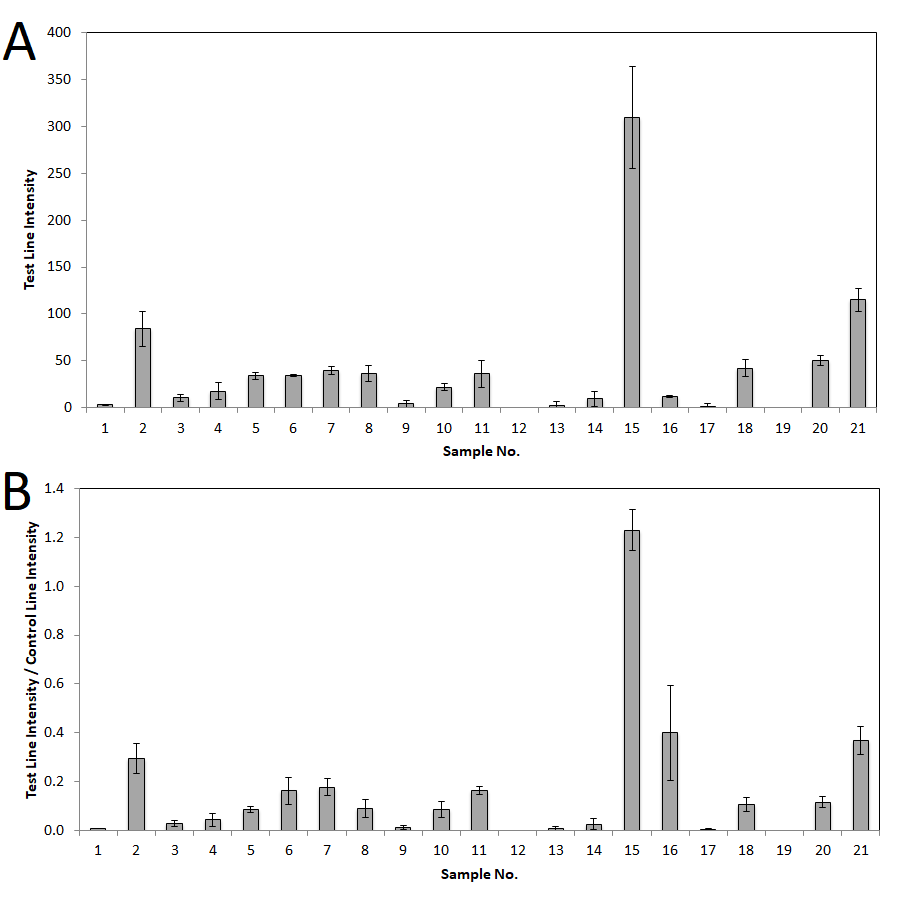


**S1 Fig.** **Twenty-one serum and plasma samples in the mixed IgG titer HSV-1/2 panel were tested using the HSV2 nanophosphor LFA.** LFA strips were then imaged on the FluorChem Platform and analyzed with ImageJ. (A) The average test line intensity values and (B) the average relative intensity values (T/C; test line intensity/control line intensity) were plotted for each member of the panel; n=3; average ± 1 standard deviation.
