# Supplementary material for: Evaluation of a nanophosphor lateral-flow assay for self-testing for herpes simplex virus type 2 seropositivity
Source: PLoS One. 2019 Dec 10;14(12):e0225365. doi: 10.1371/journal.pone.0225365 (PMC6903713; doi:10.1371/journal.pone.0225365)
Supplement: S2 Fig — FluorChem Platform images of HSV-2 PLNP LFA strips applied to panel member 16 diluted 20-fold (left) or 3.5-fold (right). Running the sample at a higher dilution reduced the sample’s viscosity, increasing reporter mobility and downstream binding at the CL. (DOCX) [file pone.0225365.s002.docx]

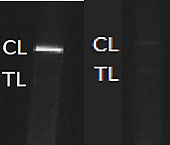


**S2 Fig.** **Sample matrix for panel member 16 had an unusual level of interfering components that adversely affected the assay.** FluorChem Platform images of HSV-2 PLNP LFA strips applied to panel member 16 diluted 20-fold (left) or 3.5-fold (right). Running the sample at a higher dilution reduced the sample’s viscosity, increasing reporter mobility and downstream binding at the CL.
