# Supplementary material for: Evaluation of a nanophosphor lateral-flow assay for self-testing for herpes simplex virus type 2 seropositivity
Source: PLoS One. 2019 Dec 10;14(12):e0225365. doi: 10.1371/journal.pone.0225365 (PMC6903713; doi:10.1371/journal.pone.0225365)
Supplement: S3 Fig — (A) Four-parameter logistic calibration curve for the total IgG ELISA created with the online MyAssays ELISA analysis tool. The curve was generated from the raw absorbance at 450 nm of dilutions of human IgG standard (30 to 0.001 ug/mL; Arista Biologicals Inc.) run in the total IgG ELISA. The raw absorbance at 450 nm of 10,000 fold dilutions of each panel samples run in the Total IgG ELISA were plotted against the standard curve (A) to calculate the IgG concentration of sample dilutions which were then used to calculate the IgG concentration of the panel samples in units of mg/mL (B). One sample (panel member 17) had a mean absorbance value that fell above the quantifiable range of standard curve and so its IgG concentration could not be accurately measured. Therefore, we excluded panel member 17 from analyses that compared LFA CL intensity with the IgG concentration. (C) FluorChem Platform control line intensity and IgG concentration of HSV2 panel members. Panel members are specified as negative (red) and positive (green) according to both PLNP HSV2 LFA and HerpeSelect 2 ELISA IgG. The sample number is placed next to each data point. Despite the variation of IgG concentration within the panel, LFA tests showed a highly similar CL across the panel, suggesting that reporter particle and IgG concentrations are more than high enough to produce a detectable CL. (DOCX) [file pone.0225365.s003.docx]

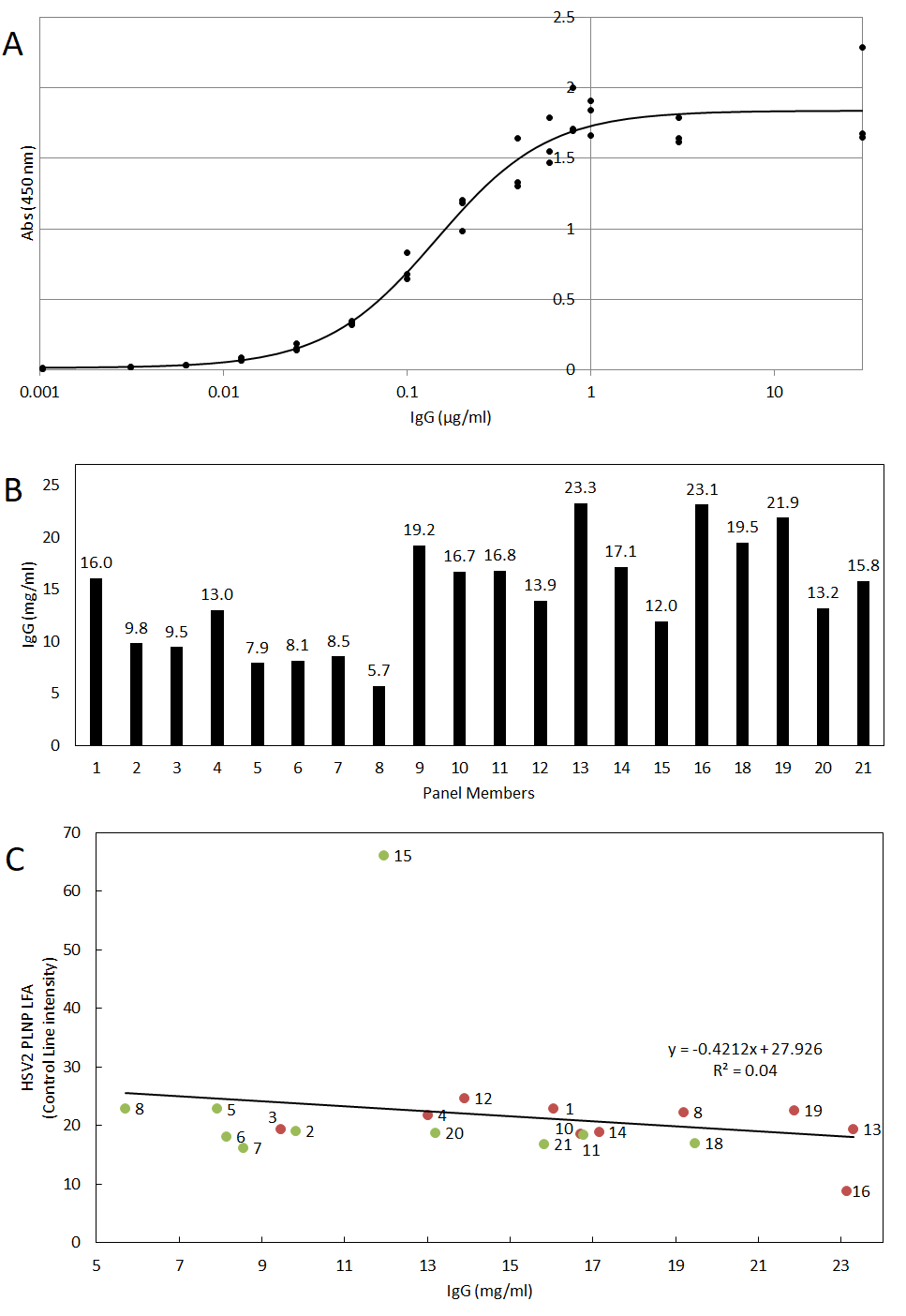


**S3 Fig. The variation of IgG concentration within the panel and its effect on HSV-2 PLNP LFA control line intensity.** (A) Four-parameter logistic calibration curve for the total IgG ELISA created with the online MyAssays ELISA analysis tool. The curve was generated from the raw absorbance at 450 nm of dilutions of human IgG standard (30 to 0.001 ug/mL; Arista Biologicals Inc.) run in the total IgG ELISA. The raw absorbance at 450 nm of 10,000 fold dilutions of each panel samples run in the Total IgG ELISA were plotted against the standard curve (A) to calculate the IgG concentration of sample dilutions which were then used to calculate the IgG concentration of the panel samples in units of mg/mL (B). One sample (panel member 17) had a mean absorbance value that fell above the quantifiable range of standard curve and so its IgG concentration could not be accurately measured. Therefore, we excluded panel member 17 from analyses that compared LFA CL intensity with the IgG concentration. (C) FluorChem Platform control line intensity and IgG concentration of HSV2 panel members. Panel members are specified as negative (red) and positive (green) according to both PLNP HSV2 LFA and HerpeSelect 2 ELISA IgG. The sample number is placed next to each data point. Despite the variation of IgG concentration within the panel, LFA tests showed a highly similar CL across the panel, suggesting that reporter particle and IgG concentrations are more than high enough to produce a detectable CL.
