# Supplementary material for: Evaluation of a nanophosphor lateral-flow assay for self-testing for herpes simplex virus type 2 seropositivity
Source: PLoS One. 2019 Dec 10;14(12):e0225365. doi: 10.1371/journal.pone.0225365 (PMC6903713; doi:10.1371/journal.pone.0225365)
Supplement: S4 Fig — In the left-hand and middle test strips, the control line (immobilized anti-human IgG antibodies) captures anti-human reporter particles bearing human antibodies from the sample (No. 21 or solution of 1 mg/ml of human IgG) to result in a visible control line. The absence of a control line, as seen in the right-hand test strip, is due to the absence of human IgG antibodies in the sample (DI water); the control line cannot capture anti-human reporter particles not bearing human antibodies. This test indicates that the control line is operating properly—only when the correct sample type has been applied onto the strip; only human serum, plasma, or blood containing human antibodies will give a positive CL. (DOCX) [file pone.0225365.s004.docx]

*
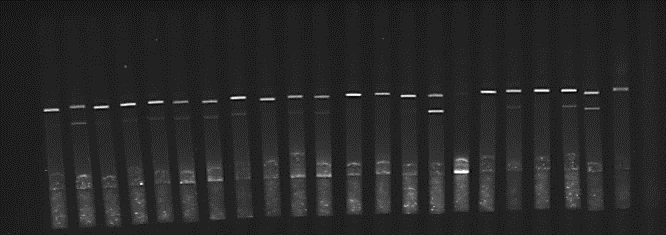

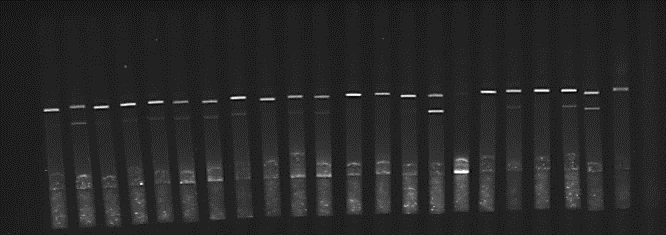
*

**S4 Fig.** **A luminescence image captured on the FluorChem Platform of three HSV-2 PLNP LFA test strips; (left) representative positive (No. 21), (middle) 1 mg/ml human IgG representative of a negative test, (right) representative inconclusive test run with DI water.** In the left-hand and middle test strips, the control line (immobilized anti-human IgG antibodies) captures anti-human reporter particles bearing human antibodies from the sample (No. 21 or solution of 1 mg/ml of human IgG) to result in a visible control line. The absence of a control line, as seen in the right-hand test strip, is due to the absence of human IgG antibodies in the sample (DI water); the control line cannot capture anti-human reporter particles not bearing human antibodies. This test indicates that the control line is operating properly - only when the correct sample type has been applied onto the strip; only human serum, plasma, or blood containing human antibodies will give a positive CL.
