# Supplementary material for: Evaluation of a nanophosphor lateral-flow assay for self-testing for herpes simplex virus type 2 seropositivity
Source: PLoS One. 2019 Dec 10;14(12):e0225365. doi: 10.1371/journal.pone.0225365 (PMC6903713; doi:10.1371/journal.pone.0225365)
Supplement: S5 Fig — Measurements obtained from iPhone 7 Plus imaging and PLNP-LFA Smartphone App analyses (upper table) and FluorChem imaging and analyses (lower table). (DOCX) [file pone.0225365.s005.docx]

**S5 Fig. Peak area measurements of the test and control lines on LFA strips run with each of the 21 panel members (n=3).** Measurements obtained from iPhone 7 Plus imaging and PLNP-LFA Smartphone App analyses (upper table) and FluorChem imaging and analyses (lower table).
