# Supplementary material for: Evaluation of a nanophosphor lateral-flow assay for self-testing for herpes simplex virus type 2 seropositivity
Source: PLoS One. 2019 Dec 10;14(12):e0225365. doi: 10.1371/journal.pone.0225365 (PMC6903713; doi:10.1371/journal.pone.0225365)
Supplement: S1 Table — For each test, a positive or negative result for each PTH202 panel member was based on the mean signal intensity (n = 3). We used the HerpeSelect 2 ELISA to determine sample positives (OD 450 ≥ 1; “an abnormal diagnosis”) and sample negatives (OD 450 < 1) and calculate the number of true-positive, true-negative, false-positive, and false-negative results for each test. (DOCX) [file pone.0225365.s006.docx]

**S1 Table. Output from STATA analysis for sensitivity/specificity and positive and negative predictive value of two rapid IgG HSV-2 tests (Fisher Sure-Vue and Focus HerpeSelect 1 & 2 Immunoblot).** For each test, a positive or negative result for each PTH202 panel member was based on the mean signal intensity (n=3). We used the HerpeSelect 2 ELISA to determine sample positives (OD 450 ≥ 1; “an abnormal diagnosis”) and sample negatives (OD 450 < 1) and calculate the number of true-positive, true-negative, false-positive, and false-negative results for each test.
